# Beyond trauma: Schema-driven psychological burden in psoriasis

**DOI:** 10.1371/journal.pone.0338947

**Published:** 2025-12-19

**Authors:** Jutta Major, András Matuz, Mehdi Moezzi, Zsuzsanna Lengyel, Béla Birkás, Boróka Gács

**Affiliations:** 1 Department of Behavioural Sciences, Medical School, University of Pécs, Pécs, Hungary; 2 Department of Dermatology, Venereology and Oncodermatology, Medical School, University of Pécs, Pécs, Hungary; 3 Szentágothai Research Centre, University of Pécs, Pécs, Hungary; Bogomolets National Medical University: Nacional'nij medicnij universitet imeni O O Bohomol'ca, UKRAINE

## Abstract

Psoriasis is a chronic inflammatory skin disease with well-documented psychological comorbidities, yet the mechanisms linking early life experiences to its psychosocial impact remain underexplored. This cross-sectional study examined the associations between childhood trauma, early maladaptive schemas (EMSs), and psychological distress in adults with psoriasis (n = 85), other chronic illnesses (n = 85), and healthy controls (n = 85). Participants completed validated self-report measures assessing childhood maltreatment (Childhood Trauma Questionnaire–Short Form), EMSs (Young Schema Questionnaire–Short Form), and symptoms of depression, anxiety, and stress (Depression Anxiety Stress Scale). Statistical analysis revealed that, relative to healthy controls, the psoriasis group endorsed higher Emotional Deprivation, Insufficient Self-Control, and Emotional Inhibition schemas, consistent with enduring emotion regulation difficulties. Psoriasis patients also reported greater depression symptoms than healthy controls, and higher anxiety and stress than both healthy and chronically ill controls. Although no between-group differences emerged in retrospectively reported childhood trauma, the pattern of schema elevations suggests that difficulties in early emotional development, such as unmet emotional needs or subtle forms of neglect, may have contributed to later vulnerability, reflected at the schema rather than the trauma-report level. Overall, the findings highlight schema-level vulnerabilities in psoriasis that may underlie psychological distress and potentially contribute to symptom maintenance. Clinically, brief screening for EMSs and emotion-regulation problems in dermatological settings may support risk stratification and referral. Integrating schema-focused and stress-reduction interventions into routine psoriasis care could improve well-being and disease management.

## Introduction

Investigating the relationship between psychological processes and certain dermatological conditions falls within the scope of psychodermatology, an emerging interdisciplinary field that explores the interconnections between the skin, the nervous system, and mental health [1–4]. It builds upon the principles of modern psychosomatics [5]and Engel’s biopsychosocial model of disease [6], integrating biological, psychological, and social factors in understanding the pathogenesis, progression, prognosis, and treatment of dermatological disorders.

Psychodermatology classifies skin conditions into three major categories [7]. First, psychophysiological skin disorders (e.g., psoriasis, eczema, acne) involve a dynamic interplay between psychological factors and dermatological symptoms. Second, primary psychiatric disorders with dermatological manifestations involve psychiatric conditions that express themselves predominantly as self-inflicted skin symptoms (e.g., trichotillomania, dermatitis artefacta). Third, dermatological disorders with secondary psychiatric symptoms include skin diseases that may provoke significant psychological distress, such as anxiety or depression (e.g., vitiligo, alopecia areata).

Within this framework, psoriasis has received growing attention as a prototypical psychophysiological skin disorder, in which psychological stress and skin symptoms interact [7,8]. Although psoriasis is a multifactorial disease with a genetic component, environmental and psychological factors are known to contribute to its onset and progression [9]. It has been linked to elevated stress levels [10,11], impaired emotion regulation [12,13], and increased prevalence of psychiatric comorbidities, including depression, anxiety, and suicidal ideation [14–16]. Despite the clinical significance of these associations, psychological aspects of psoriasis are often underdiagnosed and undertreated [17].

The neuro-immuno-cutaneous system (NICS) [18] may be a model to explain how psychological stress can impact the skin through immune modulation, hormonal changes, or disruption of the skin barrier [19]. Three categories of stressors relevant to dermatology have been identified: those arising from the disease itself (e.g., visibility, stigma), major life events (e.g., job loss, interpersonal conflict), and traumatic life experiences (e.g., childhood abuse or neglect) [20].

Recent studies have begun to explore how childhood trauma may be particularly relevant in the development of psychophysiological skin conditions such as psoriasis [21,22]. For instance, Besiroglu and colleagues [21] found that patients with so-called ‘psychosomatic’ skin diseases (e.g., psoriasis, alopecia areata, vitiligo) reported significantly higher levels of emotional neglect than those with ‘non-psychosomatic’ conditions or healthy controls. Likewise, Cansel and colleagues [22] reported that 68% of patients with chronic urticaria had a history of childhood trauma, with emotional abuse especially prevalent. These findings support the notion that chronic skin conditions, such as psoriasis, may be influenced not only by current stress but also by early adverse experiences [20]. Such trauma may disrupt emotional regulation and self-perception, contributing to long-term psychological vulnerability [23–25].

**Fig 1:**
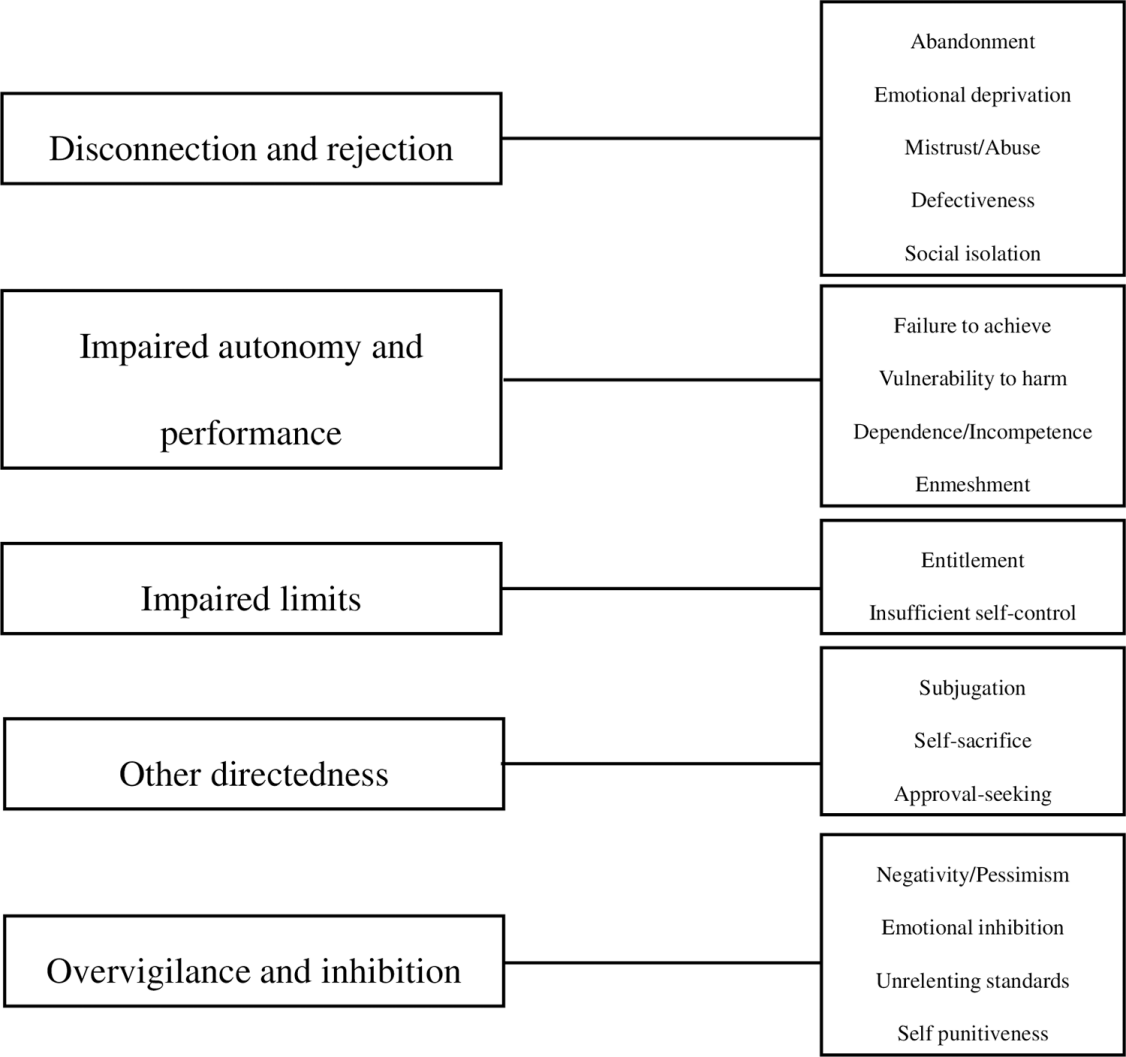
Schema domains and EMSs [28].

The term schema is widely used in psychology to describe deep cognitive structures that influence how individuals perceive, interpret, and respond to experiences [26]. Schemas act as mental filters or lenses through which current information and emotions are processed [27]. The concept of early maladaptive schemas (EMSs) refers to enduring, self-defeating cognitive-emotional patterns formed in response to unmet emotional needs during childhood [28]. Shaped by early trauma, neglect, dysfunctional caregiving, or adverse environments, these schemas can distort how individuals perceive themselves, others, and the world [28]. Young and colleagues [28] originally identified 18 EMSs grouped into five broad schema domains (Fig 1), which may contribute to persistent emotional and behavioural difficulties throughout the lifespan.

Numerous studies have confirmed the relationship between EMSs and various psychiatric disorders and symptoms, including depression [29], anxiety [30], personality disorders [31], eating disorders [32], ADHD [33], and substance use disorders [34]. Notably, EMSs have also been shown to mediate the relationship between childhood trauma and adult psychopathology, offering a psychological mechanism that could explain how early adverse experiences shape later emotional functioning [35,36].

Although empirical research on EMSs in dermatological populations remains limited [37], the high levels of psychological distress observed in patients with psoriasis [14–16] suggest that maladaptive schemas may contribute to their psychological burden. Examining the role of EMSs in this group may thus yield important insights into the mechanisms underlying their mental health challenges and guide the development of more targeted, psychologically informed interventions.

This study investigated the association between traumatic childhood experiences, early maladaptive schemas (EMSs), and psychological distress in patients with psoriasis, compared to individuals with other chronic illnesses and healthy controls. Based on previous studies [21,22], we hypothesized that psoriasis patients would report a higher prevalence of childhood trauma, particularly emotional abuse and emotional neglect. Furthermore, we expected them to show stronger endorsement of EMSs and greater psychological distress (i.e., symptoms of depression, stress, and anxiety) relative to the control groups. By examining these relationships, our research aims to deepen the understanding of the psychological factors that may contribute to the onset and progression of psoriasis. Additionally, the findings could inform the development of targeted interventions to improve patient outcomes.

## Materials and methods

### Data collection and participants

All participants completed self-administered questionnaires. The clinical group consisted of patients enrolled from a psoriasis outpatient clinic at a university-affiliated dermatology department. Members of the two control groups were recruited via various online platforms. Participants were divided into three groups: a clinical group diagnosed with psoriasis; a chronic illness control group comprising individuals with other chronic medical conditions; and a healthy control group. Data were collected between February 1, 2024, and November 30, 2024. The research was approved by the Hungarian Medical Research Council (BM/24243-1/2023) and was conducted in accordance with the Code of Ethics of the World Medical Association. Written informed consent was obtained from all subjects involved in the study. Inclusion criteria were being over 18 years of age, and for the psoriasis group, having a verified medical diagnosis. Exclusion criteria included current severe psychiatric disorders, cognitive impairment, or inability to provide informed consent. Participants in the healthy control group self-reported no chronic medical conditions.

### Measures

#### Early maladaptive schemas (YSQ-SF 3).

Early maladaptive schemas (EMSs) were assessed using the short form of the Young Schema Questionnaire, version 3 [38,39]. This self-report questionnaire comprises 90 items designed to evaluate 18 EMSs. Participants respond on a 6-point Likert scale, ranging from “1” (“completely untrue of me”) to “6” (“describes me perfectly”), with higher scores reflecting stronger dysfunctional beliefs. In our sample, the internal consistency of the schema scales ranged from α = .60 (Entitlement) to α = .89 (Social isolation, Defectiveness).

#### Childhood traumatization (CTQ-SF).

Childhood maltreatment was measured using the short form of the Childhood Trauma Questionnaire [40], a retrospective self-report instrument. It assesses five dimensions of childhood abuse: emotional neglect (EN), emotional abuse (EA), physical neglect (PN), physical abuse (PA), and sexual abuse (SA). The validated Hungarian version [41] contains 27 items (25 clinical and two validity items) scored on a 5-point Likert scale, where “1” indicates “never true” and “5” signifies “very often true.” The internal consistency of the subscales in our sample was: EN (α = .90), EA (α = .86), PN (α = .69), PA (α = .87), and SA (α = .96).

#### Psychological distress (DASS-21).

Psychological distress was assessed with the brief version of the Depression Anxiety Stress Scale [42,43]. This self-report questionnaire evaluates negative emotional states (depression, anxiety, and stress) experienced during the past week. Participants rate the extent to which each symptom applied to them on a 4-point Likert scale, where “0” denotes “did not apply to me at all” and “3” indicates “applied to me very much, or most of the time.” The internal consistency of the three subscales in our sample was as follows: Depression (α = .91), Anxiety (α = .79), and Stress (α = .85).

### Data an alyses

All statistical analyses were conducted using Jamovi, version 2.3.21.0 [44]. Descriptive statistics were computed for all variables. Group differences in demographic variables were examined using t-tests and chi-square tests. Age and gender were entered as covariates in all subsequent analyses to control for potential confounding effects.

A multivariate analysis of covariance (MANCOVA) was performed with early maladaptive schemas, DASS-21 subscales, and CTQ-SF subscales (emotional abuse and emotional neglect) as dependent variables. Given the significant multivariate effect of group, follow-up univariate ANCOVAs were conducted for each psychological variable. As the MANCOVA controlled for family-wise error, no additional correction for multiple comparisons was applied. For significant ANCOVA results, post hoc pairwise comparisons with Bonferroni correction were performed to identify specific group differences [44].

## Results

The final sample consisted of 255 participants (85 in each group). The control groups were matched to the clinical group in terms of sample size and gender distribution (χ²(2) = 1.07, p = 0.58). The clinical group included individuals diagnosed with psoriasis, while the chronic illness control group comprised participants with other chronic medical conditions. Most participants in this group had either one (58%) or two (27%) chronic illnesses, most commonly hypertension (29%), allergies (14%), diabetes (11%), musculoskeletal disorders (10%), and cardiovascular diseases (9%).

There was a statistically significant age difference between the groups: psoriasis patients (M = 46.11, SD = 15.35) were significantly older than healthy controls (M = 39.82, SD = 13.84; t(252) = 2.54, p < .05), but younger than chronically ill controls (M = 52.20, SD = 18.71; t(252) = –2.47, p < .05). Age was therefore included as a covariate in all subsequent analyses. Exploratory χ² analyses were conducted to assess potential group differences in socioeconomic indicators (place of living and educational attainment). Although both tests reached statistical significance, follow-up residual analyses showed that the differences were limited to a small number of specific categories and did not indicate systematic socioeconomic imbalance across groups. Demographic and clinical characteristics of the sample are summarized in Table 1.

**Table 1:** Demographic and mental health characteristics of participants.

Variable		Psoriasis (n = 85)		Chronic illness (n = 85)		Healthy control (n = 85)	
		n	%	n	%	n	%
Sex	Male	22	9	22	9	17	7
	Female	63	25	63	25	68	27
Age (years)	Mean (SD)	46.11 (15.35)		52.2 (18.71)		39.82 (13.84)	
Marital status	Single	19	7	13	5	23	9
	In a relationship	13	5	11	4	18	7
	In domestic partnership	12	5	2	1	8	3
	Married	31	12	44	17	31	12
	Divorced	4	2	8	3	4	2
	Widowed	5	2	7	3	1	1
	Prefers not to answer	1	0	0	0	0	0
Place of living	Village	16	6	9	4	10	4
	Small town	24	9	12	5	15	6
	Large city	21	8	19	7	24	9
	Capital city	24	9	45	18	36	14
Highest level of education	<8 years primary school	0	0	0	0	1	0
	Completed 8 years primary	2	1	1	0	0	0
	Skilled worker qualification	9	4	2	1	1	0
	High school diploma	33	13	23	9	26	10
	Bachelor’s degree	18	7	25	10	22	9
	Master’s or higher	23	9	34	13	35	14
Ever consulted a mental health professional	Yes	28	11	33	13	43	17
	No	57	22	52	20	42	16
Currently receiving psychological help	Yes	10	4	15	6	10	4
	No	75	29	70	27	75	29
Psychopharmacological medication	Currently taking	11	4	16	6	7	3
	Taken previously	7	3	4	2	1	0
	Never	67	26	65	25	77	30
Problematic substance use (self/family)	Affected (ongoing)	1	0	2	1	3	1
	Occurred in family	32	13	30	12	34	13
	No	49	19	47	18	44	17
	I do not know	2	1	2	1	4	2
	Prefers not to answer	1	0	4	2	0	0
Duration of disease (psoriasis only)	Less than a year	3	4				
	1–3 years	10	12				
	3–5 years	6	7				
	5–10 years	14	16				
	10–20 years	8	9				
	More than 20 years	44	52				
Regular medication for chronic skin condition	Yes	33	39				
	No	52	61				

Note. Duration of disease and regular medication apply only to the psoriasis group.

As shown in Table 1, participants were predominantly female across all groups, and the majority were married or living in larger cities. Educational attainment was somewhat higher in the healthy control group compared to the two clinical groups. Among psoriasis patients, more than half reported disease duration longer than 10 years, and 39% were receiving regular medication for their skin condition.

Descriptive statistics for all psychological variables are presented in Table 2. Across groups, mean YSQ-S3 scores were generally low to moderate, with the psoriasis group tending to score slightly higher on emotional and self-regulation schemas such as Emotional Deprivation, Insufficient Self-Control, and Emotional Inhibition. On the DASS-21, psoriasis patients also reported higher levels of Depression, Anxiety, and Stress relative to both control groups.

**Table 2:** Descriptive statistics for YSQ-S3, CTQ-S3, and DASS-21 scales by group.

Variable	Psoriasis	Chronically ill	Healthy control	Cronbach’s α
	M (SD)	M (SD)	M (SD)	
**YSQ-S3 Scales**				
Emotional Deprivation	2.36 (1.28)	2.17 (1.15)	1.89 (0.99)	0.85
Abandonment	2.53 (1.20)	2.46 (0.98)	2.35 (0.83)	0.75
Mistrust/Abuse	2.34 (1.25)	2.32 (0.94)	2.16 (0.86)	0.78
Defectiveness	1.78 (1.05)	1.62 (0.99)	1.52 (0.74)	0.89
Social Isolation	2.60 (1.46)	2.34 (1.15)	2.27 (1.21)	0.89
Failure to Achieve	1.95 (0.99)	1.85 (0.90)	1.76 (0.93)	0.85
Vulnerability to Harm	2.13 (1.08)	2.02 (0.97)	1.83 (0.80)	0.80
Dependence/Incompetence	1.72 (0.79)	1.66 (0.81)	1.61 (0.75)	0.76
Enmeshment	1.87 (1.04)	1.80 (0.74)	1.85 (0.75)	0.69
Entitlement	2.68 (0.91)	2.78 (0.82)	2.64 (0.85)	0.60
Insufficient Self-Control	2.62 (1.10)	2.36 (0.87)	2.30 (0.85)	0.73
Subjugation	2.12 (1.13)	2.11 (0.92)	2.12 (0.89)	0.79
Self-Sacrifice	3.29 (1.31)	3.43 (1.12)	3.06 (1.07)	0.81
Approval-Seeking	2.53 (1.25)	2.56 (1.08)	2.52 (1.05)	0.83
Negativity/Pessimism	2.54 (1.32)	2.47 (1.17)	2.20 (0.87)	0.83
Emotional Inhibition	2.45 (1.21)	2.15 (0.97)	1.98 (1.01)	0.82
Unrelenting Standards	3.08 (1.33)	3.32 (1.10)	3.06 (1.12)	0.78
Self-Punitiveness	2.59 (1.00)	2.81 (1.12)	2.52 (0.87)	0.72
**CTQ-S3 Scales**				
Physical Abuse	6.60 (3.43)	6.15 (2.10)	6.04 (2.41)	0.87
Physical Neglect	7.21 (3.04)	6.87 (2.36)	6.64 (2.69)	0.69
Sexual Abuse	5.85 (2.98)	5.66 (2.42)	5.86 (2.75)	0.96
Emotional Neglect	11.66 (5.36)	10.93 (5.21)	9.68 (4.19)	0.90
Emotional Abuse	8.74 (4.70)	8.94 (4.44)	8.22 (3.34)	0.86
**DASS-21 Scales**				
Depression	5.76 (5.58)	3.86 (4.75)	3.87 (4.41)	0.91
Anxiety	4.56 (4.17)	2.91 (3.33)	2.85 (3.27)	0.79
Stress	7.80 (4.97)	5.41 (4.64)	6.58 (4.57)	0.85
DASS Total	18.13 (13.12)	12.18 (10.55)	13.29 (10.82)	–

Note. YSQ-S3 = Young Schema Questionnaire – Short Form 3; CTQ-S3 = Childhood Trauma Questionnaire – Short Form 3; DASS-21 = Depression, Anxiety, and Stress Scale – 21 items. Psoriasis = participants with psoriasis; Chronically ill = participants with other chronic medical conditions; Healthy control = participants without chronic illness.

A multivariate analysis of covariance (MANCOVA) including early maladaptive schemas (YSQ-S3), childhood trauma subscales (CTQ-S3: emotional abuse and emotional neglect), and emotional distress measures (DASS-21 subscales) as dependent variables, with age and gender entered as covariates, revealed a significant overall multivariate group effect (F(46, 454) = 1.43, p < .05). Therefore, follow-up univariate ANCOVAs were conducted for each psychological variable to examine specific group differences. As shown in Table 3, significant group effects emerged for Emotional Deprivation, Insufficient Self-Control, Emotional Inhibition, Depression, Anxiety, and Stress, all showing small-to-medium effect sizes (η² = 0.03–0.04). Post-hoc pairwise comparisons indicated that psoriasis patients scored significantly higher than healthy controls on these variables (Emotional Deprivation (t(248) = 2.47, p < .05, Cohen’s d = 0.46), Insufficient Self-Control (t(248) = 2.81, p < .05, Cohen’s d = 0.53), Emotional Inhibition (t(248) = 2.84, p < .05, Cohen’s d = 0.53, Depression (t(248) = 3.27, p < .01, Cohen’s d = 0.61), Anxiety: t(248) = 2.79, p < .05, Cohen’s d = 0.52; Stress: t(248) = 2.41, p < .05, Cohen’s d = 0.45), and higher than the chronically ill group on Anxiety and Stress (Anxiety: t(248) = 3.03, p < .01, Cohen’s d = 0.53; Stress: t(248) = 2.76, p < .05, Cohen’s d = 0.49).

**Table 3:** Group differences in YSQ-S3, CTQ-S3, and DASS-21 scores adjusted for age and gender (ANCOVA).

Variable	F	p	η²
Emotional Deprivation	3.32	0.038*	0.03
Abandonment	1.90	0.151	0.01
Mistrust/Abuse	0.45	0.640	0
Social Isolation	1.88	0.154	0.01
Defectiveness	2.37	0.096	0.02
Failure to Achieve	2.61	0.075	0.02
Dependence/Incompetence	2.02	0.135	0.02
Vulnerability to Harm	1.02	0.361	0.01
Enmeshment	0.61	0.545	0
Entitlement	0.56	0.571	0
Insufficient Self-Control	3.98	0.020*	0.03
Subjugation	0.40	0.672	0
Self-Sacrifice	1.41	0.245	0.01
Approval Seeking	1.22	0.296	0.01
Negativity/Pessimism	2.40	0.093	0.02
Emotional Inhibition	4.06	0.018*	0.03
Unrelenting Standards	2.26	0.106	0.02
Punitiveness	1.92	0.149	0.02
Emotional Abuse	1.54	0.217	0.01
Emotional Neglect	2.08	0.127	0.02
Depression	5.46	0.005**	0.04
Anxiety	5.88	0.003**	0.04
Stress	4.69	0.010*	0.03

Note. F values are derived from separate ANCOVA tests comparing the three study groups (psoriasis, chronic illness, and healthy control) on each psychological variable, controlling for age and gender. η² = partial eta squared (effect size). *p < .05, **p < .01.

In summary, compared to both control groups, individuals with psoriasis reported greater emotional deprivation, difficulties in self-control and emotional inhibition, and elevated symptoms of depression, anxiety, and stress. No significant differences were observed in childhood trauma variables across groups.

## Discussion

This study investigated the complex interrelationships between childhood trauma, EMSs, and psychological distress in psoriasis patients compared to individuals with other chronic illnesses and healthy controls. The results shed light on the psychosocial factors associated with psoriasis and underscore the need for an integrated biopsychosocial approach to its management.

While no significant differences in reported childhood trauma emerged between the psoriasis group and the control groups, three EMSs, namely Emotional Deprivation, Insufficient Self-Control, and Emotional Inhibition, were significantly more pronounced in the psoriasis group compared to healthy controls. Importantly, despite their apparent differences, all three schemas converge on a common theme: disturbances in emotion regulation [28,45].

The Emotional Deprivation schema reflects the core belief that one’s fundamental emotional needs will not be met by others [28], often arising from early environments marked by subtle emotional neglect. Individuals with heightened activation of this schema tend to expect others to be indifferent or inattentive to their emotional needs, which can lead to persistent feelings of loneliness, mistrust, and disconnection [28]. The stronger presence of this schema among psoriasis patients may suggest that, although they did not report significantly higher trauma exposure overall, they may have experienced chronic but subtle disruptions in early emotional availability. This interpretation aligns with the view that emotional deprivation, unlike overt abuse, can be difficult to recall or recognize retrospectively but still exerts a profound psychological impact [24]. In the context of psoriasis, this schema may exacerbate emotional distress by reinforcing beliefs of being unsupported or misunderstood, especially in the face of visible and stigmatizing symptoms.

The other two EMSs identified in the study, Emotional Inhibition and Insufficient Self-Control, further underscore this pattern of dysregulated affect. According to the multidimensional model of Gratz and Roemer [46], emotion dysregulation comprises deficits in awareness, acceptance, impulse control, and the ability to employ adaptive strategies. Emotional Inhibition reflects the chronic suppression of emotional expression to avoid rejection or disapproval [28]. This avoidance can lead to internalized stress, heightened psychological distress, and impaired coping [47]. In contrast, Insufficient Self-Control involves difficulty managing impulses and tolerating distress, which can compromise stress regulation and increase emotional reactivity [28,48].

Taken together, these schemas illustrate different but complementary aspects of disrupted emotion regulation. Whereas Emotional Deprivation highlights the absence of external emotional responsiveness, Emotional Inhibition and Insufficient Self-Control reveal maladaptive internal strategies for managing affect. These findings align with evidence linking emotion dysregulation to both psychiatric disorders [23,25] and psychosomatic conditions such as psoriasis [12].

Thus, our results suggest that early maladaptive schemas, even in the absence of overtly reported trauma, may serve as trait-like markers of persistent emotion regulation difficulties [45]. By shaping how individuals perceive support, inhibit or mismanage emotional expression, and cope with stress, these schemas may contribute to the onset or exacerbation of chronic inflammatory conditions. This interpretation is consistent with previous work linking early relational adversity to impaired self-regulation and heightened vulnerability to chronic stress and illness [37,49].

Consistent with existing literature [50,51], our findings demonstrate that patients with psoriasis, compared to controls, exhibit significantly higher levels of stress, anxiety, and depression. Notably, these differences also emerged when compared to individuals with other chronic illnesses, suggesting that the psychological burden associated with psoriasis may exceed that of other long-term health conditions. This heightened mental strain may reflect the unique psychosocial challenges linked to psoriasis, such as visible symptoms, stigmatization, and body image concerns, in addition to disease chronicity and lifestyle limitations [52–54].

The observed association between depressive symptoms and psoriasis aligns with growing evidence of a bidirectional relationship between the two conditions [55,56]. Depression can worsen psoriasis through behavioural and inflammatory pathways, while the psychosocial impact of psoriasis can heighten the risk of developing mood disorders.

Stress, likewise, plays a multifaceted role in the onset and exacerbation of psoriasis. According to meta-analyses by Rousset and Halioua [9], between 26% and 88% of patients with psoriasis report stress as being associated with their condition. Stressful life events have been linked to both disease onset and symptom flares [9,57]. For instance, Manolache and colleagues [57] found that over 54% of patients reported experiencing at least one stressful event (47.36% for onset, 63.51% for recurrence or extension), compared to only 19.52% of controls. Our study revealed a similar pattern with psoriasis patients reporting the highest levels of stress compared to other chronically ill patients and healthy controls as well.

The social experience of living with psoriasis can also intensify psychological distress. Patients frequently report stigma related to the visibility of their symptoms, a factor that has been positively correlated with stress levels [53]. Fear of social rejection and internalized stigma may, in turn, intensify depressive symptoms and emotional dysregulation, creating a vicious cycle. Indeed, research has shown that individuals who perceive greater stigmatization in social contexts report more severe depressive symptoms than those who do not [53].

Psychological interventions such as schema-based therapy, mindfulness-based stress reduction (MBSR), and hypnotherapy have shown promise as effective complementary treatments for chronic skin diseases [58–60]. Integrating these approaches into psoriasis care may help patients better manage stress-related flare-ups and enhance emotional resilience [58–60]. The EMSs identified in this study (Emotional Deprivation, Insufficient Self-Control, and Emotional Inhibition) could serve as specific therapeutic targets. Moreover, the elevated levels of psychological distress observed in this population further support the use of stress reduction techniques as part of comprehensive care. In practical terms, the integration of psychological screening into dermatology clinics could be achieved through brief, self-administered questionnaires assessing emotional distress or maladaptive schemas (e.g., DLQI [61], HADS [62], PHQ-9 [63], YSQ-S3 [38]), completed by patients while waiting for consultation. Positive screening results could prompt referral to a psychologist or psychiatrist for further assessment or targeted intervention. Given the constraints of dermatological practice, a collaborative care model, where dermatologists identify at-risk patients and mental health professionals provide follow-up support, appears to be the most feasible approach. Training programs and interdisciplinary communication between dermatologists and psychologists could further improve the early detection and management of psychological aspects of psoriasis [64–67].

### Limitations

This study is not without limitations; the cross-sectional design precludes causal inferences. While the elevated EMSs in the psoriasis group may reflect early relational disruptions that precede and contribute to difficulties in emotion regulation, an alternative explanation is that the experience of living with a chronic, stigmatizing condition may itself reinforce or exacerbate maladaptive schemas over time. Experiences such as social withdrawal, rejection, or the stress associated with visible symptoms may intensify schemas like Emotional Deprivation or Emotional Inhibition. Longitudinal designs will be essential for clarifying whether these patterns represent vulnerability factors, consequences of illness-related stress, or a bidirectional process.

The reliance on self-report measures introduces the potential for recall bias, particularly in assessing childhood trauma. Future research could benefit from incorporating objective measures or corroborative reports. The study was controlled for age and gender, but other factors (e.g., disease severity, treatment history) might have influenced psychological outcomes. The groups were not matched on socioeconomic or educational background. Exploratory analyses indicated that the observed differences across groups were limited in scope and largely localized to specific categories, suggesting that substantial socioeconomic imbalance is unlikely. Nonetheless, some degree of uncontrolled variance cannot be ruled out, and future studies adopting stricter matching procedures or stratified sampling could help clarify the contribution of socioeconomic factors. Finally, control participants were recruited through online platforms, raising the possibility of sampling bias due to self-selection and variation in socioeconomic characteristics.

## Conclusion

Our findings underscore the importance of a biopsychosocial approach to psoriasis management, highlighting the role of stress responsiveness and emotion regulation difficulties in linking childhood adversity, maladaptive schemas, and psychological distress. Addressing these interconnected factors through targeted interventions can foster a more holistic treatment approach and lead to better long-term outcomes for patients with psoriasis.
